# Prokineticin-Receptor Network: Mechanisms of Regulation

**DOI:** 10.3390/life12020172

**Published:** 2022-01-25

**Authors:** Roberta Lattanzi, Rossella Miele

**Affiliations:** 1 Department of Physiology and Pharmacology “Vittorio Erspamer”, Sapienza University of Rome, Piazzale Aldo Moro 5, I-00185 Rome, Italy; 2 Department of Biochemical Sciences “A. Rossi Fanelli”, CNR-Institute of Molecular Biology and Pathology, Sapienza University of Rome, Piazzale Aldo Moro 5, I-00185 Rome, Italy

**Keywords:** GPCR, prokineticin receptors, prokineticins, transcriptional and post-transcriptional regulation, post-translational regulation, alternative splicing, binding, oligomerization, inhibitors

## Abstract

Prokineticins are a new class of chemokine-like peptides that bind their G protein-coupled receptors, PKR1 and PKR2, and promote chemotaxis and the production of pro-inflammatory cytokines following tissue injury or infection. This review summarizes the major cellular and biochemical mechanisms of prokineticins pathway regulation that, like other chemokines, include: genetic polymorphisms; mRNA splice modulation; expression regulation at transcriptional and post-transcriptional levels; prokineticins interactions with cell-surface glycosaminoglycans; PKRs degradation, localization, post-translational modifications and oligomerization; alternative signaling responses; binding to pharmacological inhibitors. Understanding these mechanisms, which together exert substantial biochemical control and greatly enhance the complexity of the prokineticin-receptor network, leads to novel opportunities for therapeutic intervention. In this way, besides targeting prokineticins or their receptors directly, it could be possible to indirectly influence their activity by modulating their expression and localization or blocking the downstream signaling pathways.

## 1. Introduction

Prokineticinin 1 (PK1 or EG-VEGF, Endocrine Gland derived-Vascular Endothelial Growth Factor) and prokineticin 2 (PK2) are small proteins that are classified as novel chemokine-like proteins based on their sequence, structure, and functions [1].

PKs have an identical AVITG sequence (alanine, valine, isoleucine, threonine, glycine) at the N-terminal amino end, which is essential for their biological activity, 10 cysteine residues (Cys) that form 5 disulfide bridges and give the molecules a very compact structure, and a tryptophan residue (Trp) at position 24, which is crucial for binding to the receptors [2,3,4]. Prokineticin receptors (PKRs) are seven-transmembrane G protein-coupled receptors (GPCRs), designated PKR1 and PKR2. PKRs can signal through the G_αs_ and G_αi_ proteins, modulating adenylate cyclase, as well as through the G_q_ proteins, promoting intracellular Ca^2+^ increase and protein kinase C activation, via the phospholipase C (PLC) pathway [5].

PKs and PKRs are expressed in both the central and peripheral nervous systems [6] and in peripheral organs and tissues such as ovaries, testes, intestinal tract, heart, bone marrow, and peripheral blood, and are involved in many physiological processes such as neurogenesis, circadian rhythm regulation, gastrointestinal motility, reproduction, and angiogenesis [7]. Dysregulation of prokineticin signaling pathways may be responsible for various pathological conditions such as cancer, pain, and inflammation. Indeed, after inflammatory insults, the prokineticin system is strongly upregulated in different tissues as lymphoid organs, circulating leukocytes and hematopoietic cells, synoviocytes and dendritic cells. This contributes to induce a proinflammatory phenotype that stimulates macrophage chemotaxis and cytokine release. This maintains a positive inflammatory feedback loop that forms the basis for the development of pathological conditions. Due to their central role in inflammation and neuroinflammation, prokineticins and prokineticin receptors have been identified as potential therapeutic targets in a variety of inflammatory/neuroinflammatory diseases [8]. In addition to its role in leukocyte trafficking, PKR2 is also involved in Chagas disease, as it enables the causative pathogen, the parasite *Trypanosoma cruzy,* to invade and infect mammalian host cells [9]. Mutations in human pkr2 or pk2 genes have been associated with genetic syndromes such as Kallmann syndrome (KS) [10] and isolated hypogonatropic hypogonadism (IHH) [11].

Given the wide range of activities in which the prokineticin system is involved, it is not surprising that numerous mechanisms have been found to regulate the activities of both prokineticins and their receptors. These mechanisms include changes in prokineticin-receptor interactions in response to a variety of environmental stimuli, modulation of protein levels in specific tissues, and changes in their molecular structures.

In this review article, we provide an overview and illustrative examples of these biochemical and cellular mechanisms by which the activity of the prokineticin system can be regulated.

## 2. Prokineticin Receptors

Prokineticin receptor 1 (PKR1) and prokineticin receptor 2 (PKR2) are the two identified receptors for prokineticins [12,13,14]. They belong to family A of G protein-coupled receptors (GPCR) with two Cys residues and show similarity to the neuropeptide Y (NPY) receptor [15]. They are approximately 85% identical with sequences that appear to be highly conserved in almost all regions except the extracellular NH2 terminus, where most sequence variation is concentrated. PKRs are differently distributed in organs and tissues: PKR1 is mainly present in peripheral tissues and in the peripheral nervous system (PNS) while PKR2 is mainly abundant in the central nervous system (CNS) [7]. Upon binding to their prokineticin ligands, the receptors undergo conformational changes that lead to the activation of intracellular effectors (G-proteins or β-arrestins) and trigger signal transduction pathways that culminate in a cellular response [16,17].

### 2.1. Genetic and Splicing Variants

**Figure 1:**
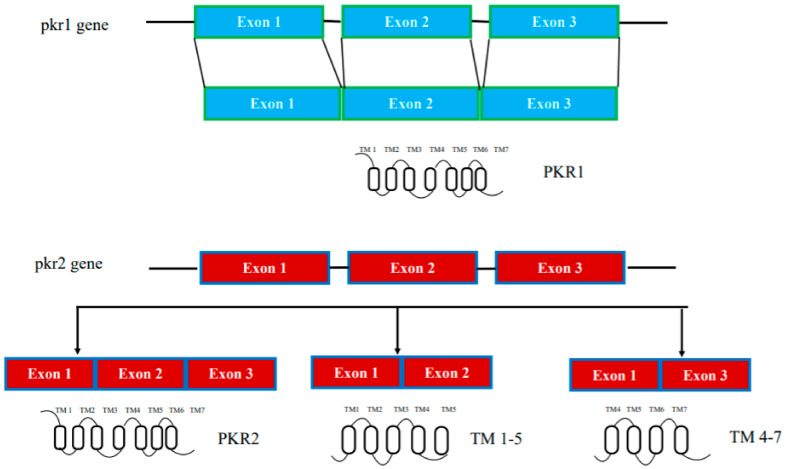
Schematic representation of the pkr1 and pkr2 gene structure.

In humans, the genes encoding prokineticin receptors are located on two different chromosomes: the pkr1 gene is located on chromosome 2 (2p13.3) and the pkr2 gene is located on chromosome 20 (20p13). In mice, the pkr genes are located on chromosome 6 and 2, respectively, and consist of three exons and two introns [15]. The first exon contains a 5′-UnTranslated Region (UTR); the second exon contains part of the 5′-UTR sequence and a region encoding the first three transmembrane domains TM1, TM2, and TM3; the third exon encodes the last transmembrane domains (TM4, TM5, TM6, and TM7) and the 3′-UTR sequence. The second intron is located at the TM3 boundary within the common DRY (Asp-Arg-Tyr) sequence (Figure 1).

The pkr2 gene is located in a region highly susceptible to mutations, as shown by linkage analysis studies and confirmed by the existence of several polymorphisms of the pkr2 gene associated with some diseases. Thus, the pkr2 polymorphism rs6053283 has been detected in idiopathic and recurrent pregnancy loss [18,19]. The presence of this rs6053283 polymorphism alters the exonic splicing enhancer (ESE) motif, which is recognized by serine/arginine-rich (SR) proteins that may be involved in the PKR2 splicing mechanism [18,19]. pkr2 gene polymorphisms have also been described in depressive and bipolar disorders and methamphetamine addiction in the Japanese population [20]. More recently, five additional polymorphisms have been found in idiopathic central precocious puberty (CPP), a syndrome characterized by premature activation of hypothalamic GnRH secretion in the absence of congenital or acquired organic lesions in the central nervous system [21]. In addition, sequence variants in pkrs genes are also associated with congenital diseases: a mutation in the pkr2 gene can lead to Kallmann syndrome, while sequence variations in pkr1 and pkr2 are associated with Hirschprung syndrome (HSCR), a syndrome characterized by the absence of enteric neural crest cells (NCC) in the distal part of the colon [10,22] (Table 1).

In addition to genetically encoded mutations, the amino acid sequences of prokineticin receptors can be altered by alternative splicing mechanisms in mRNA. One PKR2 splice variant, TM 4-7, has been identified in vivo in the rat hippocampus. It consists only of the regions spanning the last three transmembrane domains (TM) and is encoded by an mRNA lacking the second exon. TM 4-7 receptor is a functional receptor because it is activated by PK2 and is able to form heterodimers with PKR2. TM 4-7 receptor isoform appears to be upregulated under pathological conditions such as in Alzheimer’s disease (AD). In the animal model of AD, induced by intracerebroventricular (i.c.v.) injection of the b amyloid peptide toxic fragment 1–42 (Aβ_1–42_) n the rat, this PKR2 splice variant is strongly upregulated, leading to an increase in the expression ratio between TM 4-7/PKR2 in the rat hippocampus [23]. The different TM 4-7 receptor levels under physiological and pathological conditions suggest a versatile way of regulating brain functions, adding another level of complexity to the prokineticin system (Figure 1).

### 2.2. Transcriptional Regulation

Fine transcriptional regulation allows specific expression of prokineticin receptors in different tissues and in response to a variety of stimuli. In the ovaries, PKR1 is regulated by hypoxia [34]. Indeed, PKR1 appears to be strongly upregulated by a hypoxic environment in the first trimester of gestation [35]. In particular, a further PKR1 increase is induced by human chorionic gonadotropin (βhCG) between the 8th and 11th week of gestation [36]. High levels of βhCG and consequently PKR1 are observed in pathological human pregnancies, such as fetal growth restriction (FGR) and pre-eclampsia (PE) [37]. Moreover, microarray analysis and in situ hybridization demonstrated that zinc finger homeodomain family member 1 (TSHZ1), which is essential for olfactory bulb development (OB), binds and regulates pkr2 gene expression [38].

### 2.3. Structural Elements Underlying Receptors Function

Chemokine receptors are GPCRs, integral membrane proteins consisting of seven transmembrane helical segments arranged parallel to each other and packed together in a compact bundle. The extracellular side of the receptor comprises an elongated, largely unstructured N-terminal region and three connecting loops (extracellular loops, ECL1, 2 and 3) with two conserved disulfide bonds linking the N-terminus to ECL3 and ECL1 to ECL2. The cytoplasmic side of the receptor includes three additional connecting loops (Intracellular Loops, ICL1, 2 and 3) and the C-terminal region involved in G-protein coupling [39].

#### 2.3.1. Receptor-Prokineticin Interactions

Recent structures of chemokine-linked receptors confirm the two-sided model mechanism of chemokine-receptor interaction; after initial recognition of the chemokine by the N-terminus of the receptor and ECL2 (site 1), the signal is transduced by insertion of the flexible N-terminal domain of the chemokine ligand into the orthosteric pocket of the GPCR (site 2). The specific interactions with the extracellular surface of the receptor, especially with ECL2, confirm the identity of the ligand, while the insertion of the N-terminal region of the chemokine into the orthosteric TM-binding site induces conformational changes that trigger intracellular signal transduction [40].

The importance of ECL2 for ligand binding at site 1 of prokineticin receptors was first demonstrated by functional analysis of the PKR2 Q210R mutation identified in KS patients. The mutation has no effect on protein expression but strongly affects ligand binding capacity [26].

In the ECL2 of PKR2, as in all chemokine receptors, there is an aromatic residue cluster with Trp 212 localized four residues downstream of the conserved cysteine of ECL2. The crucial role of Trp at position 212 for ligand binding was demonstrated by the in vivo incorporation of p-benzoyl-L-phenylalanine, a photoactivatable unnatural amino acid, using amber codon suppression technology [41].

Prokineticin receptor site 2, the orthosteric TM-binding site identified by computational analysis, accepts the prokineticin amino sequence AVITG as well as unnatural small molecule ligands identified as prokineticin receptor antagonists [42,43]. Comparison between the binding sites of PKR1 and PKR2 TM revealed that they are completely conserved except for one residue: Valine (V) 207 in human PKR1, which is Phenylalanine (F) 198 in human PKR2. The F198V mutation in PKR2 allows obtaining a receptor capable of binding PK2β, a highly specific PKR1 ligand, more efficiently, demonstrating a role of this residue in ligand specificity [41,44,45].

#### 2.3.2. Receptor-G-Protein Interactions

The role of intracellular loops 2 and 3 in mediating interaction with G proteins was analyzed by site-directed mutagenesis.

In PKR2, the basic amino acids of the C-terminus of ICL2, which are highly conserved in GPCRs, cooperatively bind to the C-terminal five residues of the Gαq protein. This interaction was confirmed by characterization of the PKR2 mutant R164Q identified in KS patients. Indeed, this PKR2 mutant is unable to interact with Gαq, Gαi and Gα16 proteins [46].

Two parts can be identified in ICL3: a proximal and a distal sequence. The proximal sequence of ICL3 is critical for G protein coupling. A mutant obtained by deletion of the arginine 264-lysine 265-arginine 266 (RKR) sequence is normally expressed on the cell surface but shows a loss of function due to a lack of G-protein coupling. In addition, the distal region of ICL3, extending from amino acids 270 to 274, is equally important for PKR2 function. The IHH-associated PKR2 R270H mutation disrupts the interaction of PKR2 with Gαq protein but not with Gαs protein. The selective disruption may justify the biased signaling for certain mutations [11] (Table 1).

### 2.4. Maturation, Folding, and Localization

GPCRs, as well as all membrane and secretory proteins, achieve proper folding and undergo post-translational modification in endoplasmtic reticulum (ER). Subsequently, the proteins enter the Golgi apparatus that lead to their final destination within the cell. However, if a polypeptide does not complete this process correctly, the incorrect protein conformation must be detected by the quality control system and remains in the ER and/or undergoes degradation [47].

#### 2.4.1. Post-Translation Modifications: N-Glycosylation

The importance of N-linked glycosylation for the transport of PKRs was demonstrated, as has been done for numerous GPCRs, by creating a mutant with a replacement of asparagine at position 27 by glutamine. Unable to reach the membrane, the mutant shows a reduction in both Gαs and Gαq/11 signaling [48].

Mutations in ICL1 of PKR2 (R80C, R85C, and R85H) identified in patients with IHH impair N-glycosylation and localization of the receptor. PKR2 mutations R85C and R85H only slightly impair receptor function, whereas PKR2 mutation R80C leads to a marked reduction in receptor activity and exerts a dominant-negative effect on wild-type PKR2 (WT) by impairing expression of the receptor WT [27] (Table 1).

#### 2.4.2. Dimerization

Oligomerization of GPCRs can have a major impact on receptor properties, such as ligand binding selectivity, G-protein coupling, signal transduction mechanisms, and trafficking [49].

PKR2 forms active dimers in physiologically relevant cell types [50]. By heterologous expressing PKR2 in Saccharomyces cerevisiae, we investigated the mechanisms of intermolecular interaction of PKR2 dimerization. The possible involvement of three types of mechanisms was investigated: coiled coil, disulfide bridges, and hydrophobic interactions between transmembrane domains. Characterization of several deleted or site-directed PKR2 mutants suggests that dimerization occurs through interactions between transmembrane domains, particularly TMs 4 and 5. A mutant resulting from deletion of TMs 5-7 (TM 1-4) cannot associate with WT PKR2, but it can still associate with a truncated mutant lacking TMs 6-7 (TM 1-5) via a domain swapping mechanism [50]. The role of the TM5 domain in modulating PKR2 functions was demonstrated by the ability of the artificial TM 1-5 mutant to exert paradoxical gain-of-function effects when co-transfected with WT PKR2 [24]. This result was subsequently confirmed by the identification of a heterozygous frameshift mutant of PKR2 in a 3.5-year-old girl with central precocious puberty, encoding a mutant protein corresponding to TM 1-5 (Figure 1). This mutant shows no signal transduction activity in vitro, but induces distinct ligand-induced Ca^2+^ responses when it heterodimerizes with WT-PKR2 [25].

Six PKR2 mutations (R80C, L173R, W178S, G234D, V274D, and P290S) associated with KS/IHH do not reach the cell surface and are trapped in the cellular secretory pathway. Three of these mutations (W178S, G234D, and P290S) that affect trafficking are located in the transmembrane domain and likely cause the inability to form dimeric complexes [32] (Table 1).

#### 2.4.3. Binding to Accessory Proteins

Melanocortin Receptor Accessory Protein 2 (MRAP2) is an important regulator of energy homeostasis and its loss leads to severe obesity in rodents. MRAP2 was originally discovered to be an accessory protein of melanocortin receptor 4 (MC4R). Further experiments showed that MRAP2 and MC4R are not co-localized in different tissues and that MRAP2-KO or MC4R-KO mice do not show an overlapping phenotype [51]. Subsequently, other GPCRs involved in energy homeostasis have been identified as MRAP2 targets; in particular, MRAP2 significantly and specifically inhibits PKR signaling [51,52].

Specific C-terminal MRAP2 regions, conserved during evolution, are required to inhibit the activity and localization of PKRs [53]. In the presence of MRAP2, PKR2 glycosylation is largely prevented, blocking PKR2 to the ER [29].

Yeast two-hybrid screening demonstrated the interaction of snapin with the C-terminus of PKR2 (amino acids 333‣384). Snapin was originally identified as a binding protein of SNAP-25, a component of the SNARE complex, but was recently shown to also interact with the GPCR α1A-adrenoceptor [54]. The interactive motifs of PKR2 with snapin, YFK (343‣345) and HWR (351‣353), which share a similar sequence of two aromatic amino acids followed by a basic amino acid, have been mapped by GST pull-down and co-immunoprecipitation studies. The interaction between snapin and PKR2 did not affect PKR2 signaling but enhanced ligand-induced degradation, suggesting a role for snapin in PKR2 trafficking [55].

### 2.5. Internalization Recycling, or Degradation

In the past, PKRs have also been shown to be regulated by desensitization mechanisms. In in vitro experiments using Bv8, the amphibian homologous of PK2, Mollay [56] showed strong Bv8-induced tachyphylaxis during contraction of guinea pig ileum and in vivo, Cheng et al. [57] observed strong desensitization of PKRs after i.c.v. infusion of Bv8/PK2.

Receptor internalization is a mechanism used by most receptors to prevent diseases due to their excessive and dysregulated stimulation. The process begins with activation of the receptor by ligand and phosphorylation of the C-terminus of the receptor by G-protein receptor kinases (GRKs), resulting in desensitization. The phosphorylated receptor recruits β-arrestin, which attracts clathrin, resulting in internalization of the receptor into clathrin-coated vesicles. The receptor and ligand are recycled back to the cell membrane or transported to the lysosome for degradation [58].

Like many GPCRs, PKRs undergo rapid ligand-induced endocytosis followed by recycling to the plasma membrane after binding and activation by prokineticins. The mechanism underlying the internalization of PKRs is GRK2- and clathrin-mediated and independent of arrestin, as shown by Yin [17] in human embryonic kidney (HEK) cells transfected with green fluorescent protein (EGFP)-tagged PKR2 and stimulated with PK2. Indeed, in these cells, after PK2 stimulation, PKR2 recruits an unknown protein phosphorylated by GRK2 to guide the receptor to clathrin-coated pits and induces endocytosis, and on the other hand, induces Gα- and Gβγ-dissociations. The Gβγ subunit activates PLCβ and phosphorylates ERK1/2 via MEK1/2 [17].

Nevertheless, a recent paper [59] used the bioluminescence resonance energy transfer method (BRET) to investigate the association of PKRs with β-arrestin isoforms. This revealed that both receptors form stable BRET-emitting complexes with β-arrestin 2 but not with β-arrestin 1, indicating strong selectivity for the former. This is consistent with the presence of a β-arrestin 2 phosphosite signature at the C-tail sequence of PKR2, which has also been identified in other group A GPCRs such as 2-adrenoids and opioid receptors [59].

## 3. Prokineticins

Prokineticins comprise a series of proteins found in various species along the evolutionary scale from invertebrates to mammals. The history of this peptide family began in 1999 when a small protein was isolated from the skin secretion of the frog Bombina Variegata and named Bv8 to indicate its origin and molecular mass of 8 kDa [56]. Homologues of Bv8 have been found in the skin secretions of other amphibians such as Bombina bombina, Bombina orientalis, and Bombina maxima [60], in lizards, in Takifugu fish [2] and also in the venom of the black mamba snake, where it is referred to as MIT (Mamba Intestinal Toxin) [61]. Later, in 2001, two different groups [62,63] identified two human proteins called prokineticin 1 (PK1) and prokineticin 2 (PK2), respectively, because, like Bv8 and MIT, they can induce contraction of the ileum [56]. These proteins have only 44% amino acid identity. PK1, which consists of 86 amino acids and is closely related to MIT, is also called EG-VEGF [58] because it is selectively expressed in human steroidogenic organs and shows a similar effect to VEGF. PK2, which consists of 81 amino acids, is more related to Bv8. These proteins have also been found in other mammals such as mice, rats, cattle, and monkeys and the genomic structure and protein sequence have been determined [7]. Prokineticins bind and activate both PKR1 and PKR2 with the same high affinity [12,13,14]. The non-mammalian homologs, Bv8 and MIT, show PKR affinity that is an order of magnitude higher than that of human PKs [7].

### 3.1. Genetic and mRNA Splice Variants

The pk1 gene maps to regions of human chromosome 1p13.1 and mouse chromosome 3. The gene is organized into three exons; the first exon encodes 19 residues comprising 14 amino acids of the signal peptide and the first 5 amino acids of the mature protein corresponding to the AVITG sequence. The second and third exons encode the C-terminal region of the protein, which contains 10 conserved cysteine residues.

**Figure 2:**
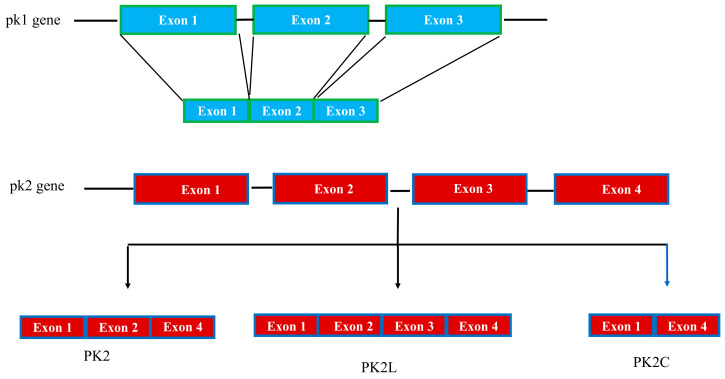
Schematic representation of the pk1 and pk2 gene structure.

The pk2 gene is located on human chromosome 3p21.1 and mouse chromosome 6 and consists of four exons. The gene organization of PK1 and PK2 is similar, except for the presence of an additional exon in the pk2 gene [64], which corresponds to the third exon and allows the production of different prokineticin isoforms by alternative splicing. The mouse pk2 gene (accession number: XP_011237860) produces four alternative splicing products. One splice product of 81 amino acids encoded by exons 1, 2, and 4 is the canonical PK2 isoform. Another PK2 isoform of 102 amino acids, called PK2L, is encoded by exons 1, 2, and 4 and generates a shorter form of PK2, called PK2b, by proteolytic cleavage that selectively binds PKR1 and induces reactions other than PK2 [44,45,65]. A third PK2 transcript, consisting of exons 1 and 2 and part of intron 2, encodes a truncated PK2 isoform of 72 amino acids that has been isolated in mouse brain and lacks both the basic section and the COOH-terminal part [64]. PK2C, encoded by exons 1 and 4, encodes a 65 amino acid protein that has recently been characterized [66] (Figure 2).

The pks genes are subject to high susceptibility to mutation. A polymorphism in pk1 (rs7513898) has been identified in association with idiopathic pregnancy loss [18]. A second polymorphism identified in the pk2 gene (rs6782813) in the 3′-UTR region is strongly associated with obesity [67].

### 3.2. Transcriptional and Post-Transcriptional Regulation

Prokineticins are ubiquitous and regulated at the transcriptional level. The pk1 and pk2 genes, similar to the VEGF gene, possess a hypoxia-induced factor one (HIF-1) binding site and their expression is induced by hypoxia [63,68]. In the reproductive tract, PK1 expression can be positively regulated by estrogen, progesterone, follicle-stimulating hormone (FSH), and human chorionic gonadotropin (hCG) [69,70]. Maternal hyperinsulinemia increases decidual PK1 expression, which could lead to impaired invasion of extravillous trophoblasts in the first trimester of pregnancy and thus a risk of pregnancy loss [71]. This insulin-induced PK1 upregulation is enhanced by dihydrotestosterone but not by testosterone [72]. PK1 expression, which is also modulated by peroxisome proliferator-activated receptor gamma (PPARγ), may mediate some of the effects of PPARγ in placental development [73].

The promoters of the Pk2 gene are extremely conserved evolutionarily, from amphibians to mammals, and have a highly conserved putative transcription factor binding site for AP-1, NF-κB, and NFAT [74]. In the olfactory bulb, the pk2 gene is upregulated by Neurogenin1 and Mash1, as well as by the circadian rhythm genes Bmal and Clock, while it is downregulated by the homeobox transcription factors (distal-less homeobox 1 and 2) [75,76]. In endothelial cells, the transcription factor TBX20 upregulates the expression of PK2, which promotes angiogenesis in an autocrine manner by binding PKR1 [77]. In monocytes and in the nervous system after nerve injury or inflammation, granulocyte colony-stimulating factor (GCSF) induces PK2 expression [34] through STAT3 activation [78].

At the post-transcriptional level, prokineticin genes are regulated by microRNAs (miRNAs), small non-coding RNAs that play a variety of crucial roles in various physiological and pathological processes [79]. PK1 expression is repressed in rat ovarian granule cells by miR-28-5p, which binds to the 3′-UTR of the pk1 gene. Overexpression of PK1 promotes cell proliferation activity, but this effect is abolished by co-transfection with miR-28-5p. PK1 also promotes polycystic ovary syndrome (PCOS) pathogenesis via the PI3K/AKT/mTOR pathway, making miR-28-5p a potential therapeutic target for this pathology [80]. miR-346 and miR-582-3p, binding sites on the 3′-UTR of pk1, downregulate PK1-induced trophoblast invasion by suppressing MMP 2 and MMP 9 [81]. The miR-200 family was predicted to target the pk1 5′-UTR. In preeclampsia patients, higher miR-200a expression reduced PK1 levels, suppressed primary cilia formation, and inhibited trophoblast invasion. Thus, miR-200a could be explored as promising miRNA biomarkers and therapeutic targets in preeclampsia, pregnancy complications and insufficient trophoblast invasion during placental development [82]. In conclusion, microRNAs may become novel therapeutic targets for PK1-related obstetric disorders.

PK2 miRNA regulation has been demonstrated in patients with Kallmann syndrome and pk2 gene mutation (c.223-4C > A). The mutant transcript contains an intron sequence of 192bp, which is a specific target of hsa-miR-3195. The expression level of miR-3195 is variable in different subjects and determines the individual expression level of the PK2 mutant, justifying the different phenotypes of patients with PK2 c.223-4C > A mutation [83].

### 3.3. Structural Elements Underlying Prokineticin-Receptor Interactions

Structural analysis of MIT [84] and Bv8 [85] by NMR revealed that all members of the AVITG family share the colipase fold. Colipases are proteins that activate the enzyme lipase, causing a conformational change. Lipase-colipase binding allows the lipase lid to move to the active site, making it accessible to the substrate [86]. This colipase fold is found in a variety of proteins, such as thick heads, proteins involved in embryonic head development, and protease inhibitors [87]. This fold consists of two similar subdomains connected by disulfide bonds. Each subdomain has a central antiparallel, three-stranded β-fold. The resulting structure is in the shape of four fingers, with the outwardly projecting loops forming surface interactions. This structure, which is very compact and stable, shows considerable resistance to heat and surface denaturants. Some charged residues are buried inside the molecules, while some hydrophobic residues are on the surface. The prokineticins and colipases, although characterized by a common fold, exhibit very limited sequence identity and a consistent charge distribution on electrostatic surfaces. This explains why the two classes of proteins have different functions: Prokineticins do not stimulate pancreatic lipase activity and colipase does not contract ileal smooth muscle. This is an interesting case of functionally divergent protein evolution from a common ancestral fold [84,85,86,87].

All members of the prokineticin family, both mammalian and non-mammalian, share the highly conserved amino-terminal AVITG sequence, which protrudes from the highly folded molecule and plays a key role in biological activity. Indeed, substitutions, deletions, and insertions at the N-terminus have produced variants of Bv8 that exhibit reduced agonist activity and sometimes antagonist activity [7,88,89].

Substitution of the first N-terminal Ala residue with methionine (Met) or addition of a Met at the N-terminus results in compounds that act as antagonists because they inhibit PKR activation [88]. Deletion of the first Ala residue (des-Ala-Bv8) from the N-terminus results in a compound that has 5 times lower affinity for PKRs and is 20 times less potent in inducing hyperalgesia compared to Bv8. Deletion of the first two Ala-Val residues (des-Ala-Val Bv8) from the N-terminus results in a compound that is still able to bind PKRs but cannot activate them. It has no biological activity both in vitro and in vivo, but can antagonize Bv8-induced hyperlgesia [89]. Moreover, the missense mutation described in KS patients, which causes a substitution of glycine in the N-terminal AVITG sequence, leads to a complete loss of function [33].

The C-terminal domain also plays an essential role in biological activity. Indeed, peptides with mutations in some cysteine residues in the C-terminus or lacking the carboxyl domain or with the N-terminus fused to the carboxyl terminal of colipase or thickhead are not biologically active [88]. In some sense, the mutants C34Y and C46Y described in KS, characterized by the substitution of a cysteine residue by tyrosine, show very low activity [28]. The hydrophobic tryptophan residue at position 24 (Trp24) is exposed on the surface of the prokineticin protein family and is crucial for the affinity and activity of PKRs. Substitution of Trp24 by Ala produces an analog (Ala24-Bv8) that behaves as an agonist or antagonist depending on the dose. At high doses, it acts as an agonist in both mice and rats, producing thermal hyperlagesia and tactile allodynia [4]. In contrast, at low, ineffective doses, it acts as an antagonist that reduces Bv8-induced hyperalgesia for many hours. Moreover, Ala24-Bv8 also inhibits hyperalgesia induced by inflammation, a condition in which PK2 is strongly upregulated [90]. The anti-hyperalgesic effect is attributed to central mechanisms involving both an increase in ss-endorphin levels in the midbrain and hypothalamus and inhibition of the endogenous inhibitory descending pain pathway [4].

### 3.4. Post-Translational Modifications

The specificity of cellular responses is influenced by the ability of ligands to induce different GPCR-activated downstream effectors, but also by the kinetics and spatial compartmentalization of pathway activation. In particular, binding to extracellular matrix components such as heparin sulfate proteoglycans may be a means of controlling the bioavailability of various secreted molecules, including basic fibroblast growth factor and VEGF [91]. It has been demonstrated that EG-VEGF binds with high affinity to heparin-Sepharose because it contains a consensus sequence of heparin-binding proteins (XBBXBX motif, in which B is a basic amino acid) [68]. PK2 and PK2β both bind to heparin-Sepharose, but PK2β has a higher affinity. In fact, the PK2β-encoding mRNA contains exon 3, which encodes a 21-amino acid insertion containing a consensus sequence of heparin-binding proteins. Using the heterologous yeast expression system, it has been demonstrated that heparin does not affect PK2 activity, but negatively affects PK2β activity, particularly on PKR1 [45].

## 4. Natural and Pharmacological Inhibitors

### 4.1. Parasites

PKR2 has been described as a novel receptor that enables the pathogenic parasite Trypanosoma cruzy to invade and infiltrate mammalian host cells by binding a LamG domain of Tc85, a glycoprotein belonging to the trans-sialidase family [92]. Demonstrating the ability of the LamG domain to activate PKR2, we showed that PKR2 induces strong ERK, NFAT, and STAT3 phosphorylation in CHO mammalian cells and in mouse dorsal root ganglia explants after LamG binding [9]. L173R is the most common PKR2 mutation reported in KS, and it is a founder mutation. It has been shown that in cells co-expressing the PKR2-L173R mutant and PKR2-WT, LamG cannot induce signal transduction, suggesting that the L173R mutation in heterozygosity may provide a selective advantage in protecting against Trypanosoma cruzy infections [9].

### 4.2. Antagonists

Given the role that the prokineticin system plays in various inflammatory and neuroinflammatory diseases, great efforts have been made to search for molecules that can block this system. Research has worked on two fronts, searching for PK2 antibodies or PKR antagonist molecules.

Both PK1 and PK2 induce proliferation, migration, and angiogenesis of endothelial cells and play important roles in tumors, particularly those characterized by heavy infiltration of neutrophils, such as colorectal cancer and non-small cell lung cancer [93,94]. In particular, a specific subpopulation of myeloid cells, termed CD11b+Gr+, has been identified in patients with these cancers. These cells, mainly composed of neutrophilic granulocytes but also macrophages and dendritic cells, infiltrate tumors and stimulate angiogenesis, which promotes tumor growth. VEGF is recognized as an essential regulator of normal and abnormal blood vessel growth, and treatment with the anti-VEGF antibody bevacizumab is now a first-line therapy for metastatic colorectal cancer, although unfortunately many cases of resistance due to tumor infiltration by CD11b+Gr+ cells have been reported. Since PK2 is highly upregulated in CD11b+Gr+ cells and associated with such resistant tumors, combined therapy with anti-PK2 and anti-VEGF antibodies could be a strategy to slow the growth of anti-VEGF resistant tumors [93].

As described earlier, substitution or deletion in the PK2 molecule resulted in compounds with antagonistic activity. However, being proteins, they are subject to enzymatic degradation, are expensive and, therefore, not very suitable for clinical use. For this reason, a number of non-peptide compounds have been synthesized in recent years [95]. These include a group of triazine-guanidine derivatives that mimic the structural features required for binding to the PKs receptor: The triazine-guanidine moiety mimics the N-terminal AVITG sequence, while the methoxybenzyl moiety of the antagonist is aligned as a tryptophan residue at position 24 [3]. These compounds are named PC (PC1/PC36) and have been pharmacologically characterized both in vitro and in vivo. Radioligand binding and BRET experiments [96] have shown that all these molecules are able to bind with high affinity to the prokineticin receptors, precisely in a specific receptor pocket located in the upper part of the TM bundle between TMs 3, 4, 5, 6, and 7, as suspected from the analysis of human PKRs [3,42].

The lead compound PC1 is the best characterized and has been found to antagonize several pathological conditions due to PK2 overexpression. PC1 reduces inflammatory [90] and neuropathic pain [97,98,99,100,101,102,103] and reduces deficits in animal models of AD [104]. In addition to PC1, PC7 has also been reported to reverse the effects of PKR in other systems. It reduces demyelination in Experimental Autoimmune Encephalomyelitis (EAE), a mouse model of multiple sclerosis [105], and it appears to be useful in the treatment of pregnancy disorders such as preeclampsia and fetal growth restriction associated with abnormal PK1 expression. Indeed, PK1 plays a central role in normal pregnancy by controlling trophoblast invasion and placental development, and the use of PC7 improves pregnancy outcome [106]. PKRA7 is [(3R)-1-(4-Fluoro-3-methoxybenzyl)-N-(9-chloro-3,4-dihydro-2H-1,5-benzodioxepin-7-ylmethyl)-N-isobutylpyrrolidine-3-carboxamide] synthesized to mimic mutated peptides of the N-terminal region of PK2 obtained by replacing an alanine residue with methionine and adding methionine at the N-terminus of PK2 (Table 2). PKRA7 acts as a PKR antagonist [107] and blocks angiogenesis and macrophage infiltration in mice transplanted with glioblastoma and pancreatic cancer, respectively [107], and significantly reduces the severity of arthritis in mice [108]. More recently, PKRA7 has also been shown to play a role in reducing the PK2-mediated inflammatory process during uropathogenic Escherichia coli (UPEC)-induced orchitis by suppressing PK2 activity [109].

## 5. Summary and Future Directions

Prokineticins and their receptors play a central role in immune and inflammatory responses, in cancer, and in various processes involved in nervous system and reproductive organ development.

**Figure 3:**
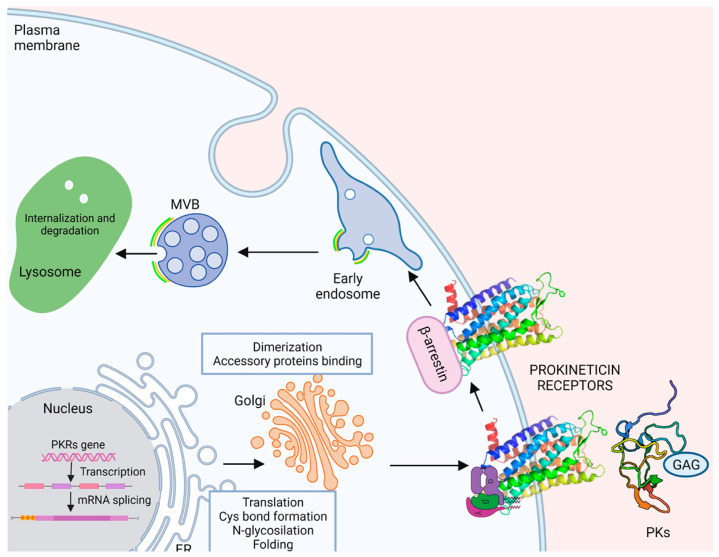
Schematic overview of regulation mechanisms of the prokineticin receptors. GAG: proteoglycans; MVB: multivesicular body.

In this review, we have attempted to elucidate the major regulatory mechanisms that contribute to the complexity of the prokineticin system and enable it to respond to various stimuli (Figure 3).

Understanding the regulatory mechanisms also leads to new possibilities for therapeutic interventions. For example, prokineticin receptors could be targeted directly or indirectly by exploring increasingly selective antagonists. The latter strategy involves modulating the prokineticin transduction system by inhibiting prokineticin receptor expression, maturation/localization, and downstream signaling pathways.

## Figures and Tables

**Table 1:** Mutations of prokineticin receptor 2, biochemical and functional characteristics, and their implications in different diseases. PL: Pregnancy loss; DD: depressive disorders; AD: Alzheimer’s disease; PP: Precocius puberty KS: Kallmann syndrome; IHH: Hypogonatropic hypogonadism.

Polymorphisms	Phatology	Mechanism	References
rs6053283	PL, DD	Alteration of the exonic splicing	Su et al., 2010 [18] Cao et al., 2016 [19]
**Splice variants**			
TM 4-7	AD	Impaired dimerization with PKR2	Lattanzi et al., 2019 [23]
TM 1-5	PP	Increase of Gα coupling capacity	Sposini et al., 2015 [24] Fukami et al., 2017 [25]
**Mutations**			
R80C	KS, IHH	Impaired PKRs N-glycosylation	Monnier et al., 2009 [26] Abreu et al., 2012 [27]
R85H	IHH, HSCR	Impaired PKRs N-glycosylation	Monnier et al., 2009 [26] Ruiz Ferrer et al., 2011 [22] Abreu et al., 2012 [27]
R85C	IHH, HSCR	Impaired PKRs N-glycosylation	Cole et al., 2008 [28] Ruiz Ferrer et al., 2011 [22] Abreu et al., 2012 [27]
R85G	IHH	Impaired Gα coupling	Cole et al., 2008 [28] Monnier et al., 2009 [26]
Y113H	KS, IHH	Impaired Gα coupling	Cole et al., 2008 [28] Zhao et al., 2019 [11]
V115M	KS	Impaired Gα coupling	Cole et al., 2008 [28]
L218P	IHH	Impaired Gα coupling	Zhao et al., 2019 [11]
R164Q	KS	Impaired Gα coupling	Cole et al., 2008 [28] Monnier et al., 2009 [26]
L173R	KS, IHH	Inability to reach the cell surface	Cole et al., 2008 [28] Monnier et al., 2009 [26] Abreu et al., 2010 [29] Libri et al., 2014 [30] Cox et al., 2018 [31]
W178S	KS, IHH	Inability to reach the cell surface	Cole et al., 2008 [28] Monnier et al., 2009 [26] Chen et al., 2014 [32] Zhao et al., 2019 [11]
S188L	KS	Impaired Gα coupling	Cole et al., 2008 [28]
Q210R	KS, IHH	Impaired ligand binding	Dodé et al., 2006 [33] Monnier et al., 2009 [26]
L218P	IHH	Impaired Gα coupling	Zhao et al., 2019 [11]
G229R	IHH	Inability to reach the cell surface	Zhao et al., 2019 [11]
E231K	IHH	Inability to reach the cell surface	Zhao et al., 2019 [11]
G234D	KS, IHH	Impaired dimerization with PKR2	Chen et al., 2014 [32] Cox et al., 2018 [31]
R248Q	KS	Impaired Gα coupling	Cole et al., 2008 [29]
T260M	IHH	Impaired Gα coupling	Monnier et al., 2009 [26] Libri et al., 2014 [30]
R268C	IHH, HSCR	Impaired Gα coupling	Libri et al., 2014 [30] Ruiz Ferrer et al., 2011 [22] Cox et al., 2018 [31]
R270H	IHH	Impaired Gα coupling	Zhao et al., 2019 [11]
V274D	KS, IHH	Inability to reach the cell surface	Libri et al., 2014 [30]
P290S	KS, IHH, HSCR	Inability to reach the cell surface	Monnier et al., 2009 [26] Ruiz Ferrer et al., 2011 [22] Chen et al., 2014 [32] Cox et al., 2018 [31]
V331M	KS, IHH	Impaired Gα coupling	Cole et al., 2008 [29] Monnier et al., 2009 [26] Libri et al., 2014 [30]
V334M	IHH	Increased ability to reach cell surface	Libri et al., 2014 [30]
R353H	IHH	Impaired Gα coupling	Zhao et al., 2019 [11]

**Table 2:** Ligand binding affinities to PKR1 and PKR2 [110,111].

Compound	PKR1 (K_i_, nM)	PKR2 (K_i_, nM)
Bv8	0.69	0.71
MIT	4.1	0.67
PK1	250	81
PK2	6.9	7.6
PK2β	34.6	>1000
PC1	72	702
PC7	18	1024
